# LINE-1 is preferentially hypomethylated within adenomatous polyps in the presence of synchronous colorectal cancer

**DOI:** 10.1186/s13148-017-0325-7

**Published:** 2017-03-09

**Authors:** Alice Chu Jiang, Lela Buckingham, William Barbanera, Amoah Yeboah Korang, Faraz Bishesari, Joshua Melson

**Affiliations:** 1 Department of Internal Medicine, Rush University Medical Center, 1717 W Congress Parkway, 10 Kellogg, Chicago, IL 60612 USA; 2 Department of Pathology, Rush University Medical Center, 600 S. Paulina Street, 1014 AAC, Chicago, IL 60612 USA; 3 Division of Digestive Diseases, Department of Internal Medicine, Rush University Medical Center, 1725 West Harrison, Suite 206, Chicago, IL 60612 USA

**Keywords:** LINE-1, Field defect, Colorectal cancer, Global methylation

## Abstract

**Background:**

Conventional tubular adenomas are frequently detected in patients undergoing average risk screening colonoscopy and are over-represented in patients who will develop colorectal cancer (CRC). Whether features of adenomas could serve as predictors of synchronous CRC is not known. Here, we investigate whether global methylation markers, including LINE-1, differ within adenomas in patients with and without synchronous CRC.

**Methods:**

Colorectal tubular/tubulovillous adenomatous polyps in the absence (P group, *n* = 45) and in the presence of synchronous CRC (PC group, *n* = 32) were identified. Global methylation and demethylation by ELISA for 5-methylcytosine (5-mC) and 5-hydroxymethyl cytosine (5-hmC), respectively, were assessed in polyps and adjacent normal non-neoplastic tissue. LINE-1 hypomethylation was assessed by pyrosequencing of bisulfite-converted DNA as well.

**Results:**

Global methylation (5-mC) showed no differences in overall methylation status in the adenomatous polyps in the two groups (5-mC relative to control %, PC group 0.117; P group 0.161, *p* = 0.148). Global hydroxymethylation 5-hmC was also not significantly different in adenomatous polyps of the PC group than in those of the P group (0.0059 vs 0.0097, *p* = 0.681). Similarly, global 5-hmC was not different between normal tissues from patients without neoplasia in comparison to those from CRC patients (0.0461 ± 0.080 vs 0.039 ± 0.159, *p* = 0.215). In contrast, adenomatous polyps of the PC group had lower levels of LINE-1 methylation compared to the adenomas in the P group (53.07 ± 4.5 vs 59.95 ± 5.4, *p* < 0.001). LINE-1 methylation was also significantly lower in the normal tissue from cancer patients compared to that from patients without any neoplasia (58.07 ± 3.78 vs 71.50 ± 6.47, *p* < 0.001).

**Conclusions:**

LINE-1 hypomethylation of precancerous adenomas correlates with the presence of synchronous CRC. Measurement of DNA hypomethylation levels of colorectal adenomas by LINE-1 could have future implications in approaches to defining CRC risk in screening programs.

## Background

Adenoma detection rates (ADR) in patients who undergo screening colonoscopy have incrementally increased over time. With improvements in endoscopic imaging modalities, it is not uncommon for ADR to approach or even exceed 50% of cases undergoing screening colonoscopy [1]. The majority of patients who participate in screening colonoscopy programs will ultimately develop an adenoma [2], [3]. Surveillance intervals for repeating follow-up colonoscopy are based on pathology features defining advanced adenomas by the presence of villous histology or high-grade dysplasia, size of polyp, or multiplicity [4, 5]. Even in patients whose initial colonoscopy yields an advanced adenoma, and thus deemed the high-risk group based on US Multi-Society Task Force (USMTF) recommendations, still the majority of these patients will not have further advanced neoplasia on surveillance colonoscopy [6, 7]. Alternative ways to define risk for concerning colorectal neoplasia could have clinical implications to better utilize colonoscopy surveillance resources.

CRC develops from both genetic and epigenetic alterations along a tumorigenesis sequence [8–11]. A type of epigenetic alteration, DNA hypomethylation of repetitive sequences [i.e., short interspersed elements (SINEs or Alu elements) or long interspersed nuclear elements (LINEs)] may predispose cells to chromosomal defects and rearrangements that result in genetic instability and elevated mutational rates [11–13]. Aberrant DNA methylation can also be measured through quantification of global DNA methylation. Global DNA hypomethylation has been found to be greater in adenomas than in normal mucosa [14]. However, as global methylation can vary with cellular function, LINE-1 hypomethylation is thought to correlate to chromosomal instability and CRC dysplasia progression [15–17]. In the normal colorectum, absent of dysplasia, LINE-1 is highly methylated. In CRC progression, LINE-1 hypomethylation is described in the precancerous adenomatous polyp stage and tracks with TNM staging of CRC [17] and poor survival [18]. Therefore, evaluation of LINE-1 methylation as a specific marker of global methylation may be a valuable tool for risk stratification of CRC development.

If a potential informative high-risk biomarker is present in adenomas associated with concomitant or synchronous CRC, this could then be considered as a potential marker to predict risk of metachronous neoplasia as well as be informative about potentially missed synchronous lesions. Here, we performed an analysis of conventional adenomas for potential markers of synchronous neoplasia based on analysis of global methylation and global hypomethylation status as well as LINE-1.

## Methods

### Patients

Formalin-fixed specimens of single representative tubular or tubulovillous polyps (largest size) and CRC from each patient were included in the analysis. Global methylation, global hypomethylation, and LINE-1 methylation status was contrasted between all groups. Comparisons between groups included adenomas with synchronous CRC versus those without. Comparative groups of non-neoplastic tissue were between normal non-neoplastic tissue in patients who underwent screening colonoscopy without neoplasia versus normal mucosa in patients with synchronous CRC (PC group).

Patients with prior colonic resections and inflammatory bowel disease, or a prior history of CRC, were excluded. Patients with in excess of five colorectal adenomatous polyps were excluded as well. The study was limited to conventional tubular/tubulovillous/villous adenomas. Sessile serrated polyps that are considered to have a distinct carcinogenic pathway were not included.

The study was conducted according to the principles of the Declaration of Helsinki. The protocol was approved by the Internal Review Board (IRB) of Rush University Medical Center (14080703-IRB01), and informed written consent was obtained from all patients.

### Specimen collection

Three 4-μm sections were cut from fixed tumor, polyp, or non-malignant tissue sections. One slide for each specimen was stained with hematoxylin and eosin (H&E). Normal tissue specimens were taken from separate tissue blocks discrete from tumor blocks and at least 1 cm away from neoplasia. Collected data included gender, race, genotype, age at diagnosis, date of diagnostic colonoscopy, additional polyps, focal high-grade dysplasia, cancer arising from adenoma, location (right or left), tumor size (cm), and tumor stage.

### DNA isolation

Using the reviewed H&E-stained slide as a guide, tumor, polyp, or non-malignant tissue was scraped from one or two slides (macrodissection) and placed in 60–200 μL lysis buffer (10 mM Tris 50 mM KC, pH 8.3, 1.0 mg/mL proteinase K). Tissue for assay was microdissected from unstained slides. Adjacent H&E-stained slides were used for pathological review to determine what area of the tissue section contained the purest tumor. Microdissection of tissue samples by pathologists yielded >90% dysplastic material in the samples. The volume used depended on the amount of tissue available from the slides. The samples were incubated for at least 6 h before methylation analysis. Proteinase activity was eliminated at the end of the digestion by a 5-min incubation at 95 °C.

### CpG island methylator phenotype (CIMP) analysis

Sodium bisulfite treatment of 10 μL of the DNA extracted as described above was performed using the Qiagen EpiTect system according to the manufacturer’s protocol. Following bisulfite treatment, which converts unmethylated cytosines to uracil, the converted DNA was amplified using the MethyLight real time. PCR was performed according to the manufacturer’s protocol. Values for each gene promoter in the classic panel, *APC*, *CDKN2*, *MINT1*, *MINT2*, *MINT31*, and *MLH* normalized to *COL2A1* amplification control were calculated by linear regression from a standard curve and expressed as a percent of M.SssI-treated (100% methylated) genomic DNA. A cut point was selected for each gene to dichotomize the data into methylated yes/no. CIMP-positive samples were those with three sixth of the gene promoters methylated.

### Global methylation

Global methylation was assessed by ELISA analysis using the Epigentek MethylFlash^TM^ Methylated DNA Colorimetric Quantification Kit (Epigentek, Farmingdale, NY). DNA concentration in the sample lysates prepared as described above was estimated by spectophotometry (NanoDrop, Thermo Fisher). The analyses were done directly on the tissue lysates. DNA was purified after bisulfite conversion with the Zymo bisulfite conversion kit. One hundred nanograms was used for each methylation analysis. Positive and negative controls were supplied with the kit. A response curve was prepared by dilution of the supplied standard. The positive control concentration was 5 ng/μL. DNA in a volume of 1–8 μL was analyzed according to the manufacturer’s procedure. The resulting absorbance was measured on a SpectraMax plate reader. Absorbance was converted to relative methylation, 5-mC% (to control), by the following formula:

$$ \mathrm{relative}\ 5\hbox{-} \mathrm{m}\mathrm{C}\% = \frac{\left(\mathrm{sample}\ \mathrm{abs} - \mathrm{neg}\ \mathrm{control}\ \mathrm{abs}\right) \div \mathrm{input}\ \mathrm{DNA}\ \left(\mathrm{ng}\right)}{\left(\mathrm{pos}\ \mathrm{control}\ \mathrm{abs} - \mathrm{neg}\ \mathrm{control}\ \mathrm{abs}\right) \times 2 \div 5\ \mathrm{ng}} $$

### Global demethylation (5-hydroxymethyl cytosine)

Two hundred nanograms was used for each hydroxymethylation analysis, performed by the same procedure used for 5-methyl cytosine detection, using primary antibodies for 5-hydroxymethyl cytosine. The observed absorbance was converted to relative methylation, 5-hydroxymethyl C% (to control), by the following formula:

$$ \mathrm{relative}\ 5\hbox{-} \mathrm{h}\mathrm{m}\mathrm{C}\% = \frac{\left(\mathrm{sample}\ \mathrm{abs} - \mathrm{neg}\ \mathrm{control}\ \mathrm{abs}\right) \div \mathrm{input}\ \mathrm{DNA}\ \left(\mathrm{ng}\right)}{\left(\mathrm{pos}\ \mathrm{control}\ \mathrm{abs} - \mathrm{neg}\ \mathrm{control}\ \mathrm{abs}\right) \times 5 \div \mathrm{input}\ \mathrm{ng}} $$

### LINE-1 methylation

LINE-1 methylation was assessed by pyrosequencing of bisulfite-converted DNA [19]. Precision of the pyrosequencing assay for LINE-1 methylation levels was previously validated for colon cancer tissue and normal colonic mucosa as previously described [20]. Ten microliters of DNA lysate from microdissected polyp, tumor, or non-malignant tissue were bisulfite converted using the Zymo EZ DNA Methylation TM Kit (Zymo Research, Irvine, CA) following manufacturer’s protocol. The converted DNA was amplified using primers. The LINE-1 retrotransposon targeted was located on 22q11-q12; genomic coordinates (GRCh38): 22:15,000,000-37,200,000; the primer sequences were based on repeat elements (locus X58075:111-358). Analysis was based on LINE-1 sequence with GenBank accession number ONS374723. After amplification, 15 μL PCR product was subjected to pyrosequencing. Sequencing was performed on a Pyromark Q24 Pyrosequencer (Qiagen), programmed with the following sequence to be analyzed: TYGATTTTTTAGGTGYGTTYGTTA. The dispensation order was GTCGATTAGTAGTCAGTCGT. The average of the relative percent C (methylated) versus T (unmethylated) at each of three CpG sites was reported. Non-CpG cytosines, which should be 100% converted, were included in each sequence to confirm complete conversion.

### Statistical analysis

Basic summary statistics were calculated for global methylation levels and other continuous variables. Binary and categorical variables were tabulated. Means for global methylation and global hypomethylation were compared by independent two-sided *t* test. Pearson’s correlation test was used to evaluate strength of association between hypomethylation of LINE-1 in polyps of the PC group and tumor tissue. Wilcoxon signed rank test was used to compare differences in median hypomethylation of LINE-1 in polyps of the PC group and tumor tissue. Analyses were performed in SPSS statistical software. Statistical significance for all analyses was deemed to be *p* ≤ 0.05.

## Results

### Study population

There were 45 patients in the adenoma and no synchronous CRC (P group) and 32 patients in the adenoma and synchronous CRC (PC group). In addition, there were eight specimens of tissue from patients with no neoplasia or history of prior neoplasia who had undergone screening colonoscopy (N group). Normal mucosa from 45 patients of the P group and normal mucosa from 32 patients of the PC group were also included in the analysis. Patient demographics of included cases are shown in Table 1. LINE-1 methylation and global hypomethylation studies were performed on all cases. Out of the 45 patients in the P group, follow-up colonoscopy was performed on 30 patients, ranging from 1 to 6 years, with a median of 5 years. None had metachronous colorectal cancer on follow-up.

**Table 1:** Characteristics of patients with polyps

Group	P (*n* = 45)	PC (*n* = 32)	Independent *t* test, *p* value
Age	60.4 ± 8.7	68.4 ± 11.0	p = 0.001
Female	38% (17)	44% (14)	p = 0.60
Right colon polyp location	55% (25)	68% (22)	p = 0.24
Polyp histology			
Tubular	84% (38)	84% (27)	p = 0.50
Tubulovillous or villous	16% (7)	16% (5)	p = 0.50
CIMP positive	11% (5)	9% (3)	p = 0.60

Right colon location is defined as proximal to the splenic flexure. CIMP positivity defined as three out of six markers methylated

*n* normal, *P* polyp without synchronous cancer, *PC* polyp with synchronous cancer

Of the 32 patients with CRC, 28 had moderate to poorly differentiated tumor tissue, 14 were stage I or tumor in situ, 8 were stage II, 7 were stage III, and 3 were stage IV. Microsattelite instability (MSI) testing was done on those that met Bethesda criteria, which included 10 of the 32 tumor tissue samples, with 2 MSI-H.

### Global methylation 5-mC analysis in polyps of patients with synchronous CRC (PC group) compared to those without synchronous CRC (P group)

**Fig. 1:**
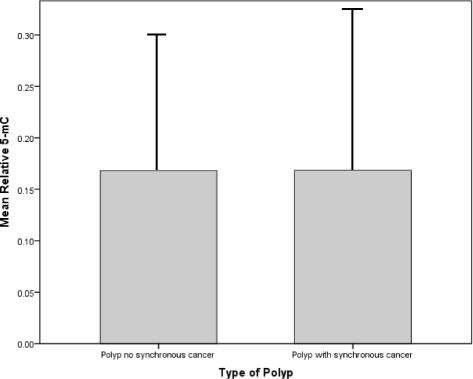
Relative percent 5-mC in tubular polyps from patients with and without cancer (*N* = 18, *N* = 22, respectively, 0.168 ± 0.413 vs 0.168 ± 0.334, *p* = 0.997 by independent *t* test)

Global methylation analysis by 5-mC showed no significant differences in overall methylation status between the two adenoma polyp groups (P and PC) (0.168 ± 0.413 vs 0.168 ± 0.334, *p* = 0.997, Fig. 1).

### Global hydroxymethylation (demethylation) in polyps of patients with synchronous CRC compared to those without synchronous CRC

**Fig. 2:**
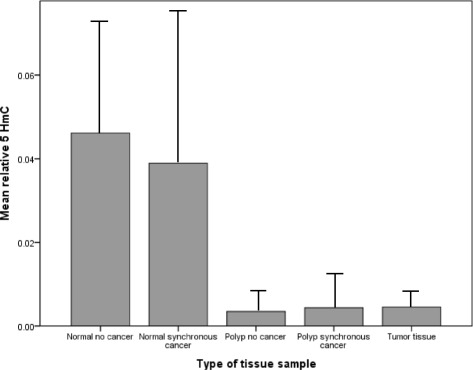
Relative 5-hmC of tubular polyps in patients without (*N* = 50) (0.0035 ± 0.0086) and with (*N* = 25) (0.0043 ± 0.0112) CRC (*p* = 0.712 by independent *t* test), compared to normal tissue from patients with (*N* = 35) and without (*N* = 17) CRC and tumor tissue. Normal tissue from patients without cancer had similar 5-hmC compared to normal tissue from patients with CRC (0.0461 ± 0.080 vs 0.039 ± 0.159, *p* = 0.215 by independent *t* test)

5-hmC was not significantly higher in the polyps from patients with cancer (PC group) than in the polyps from patients without cancer (P group) (0.0035 ± 0.0086 vs 0.0043 ± 0.0112, *p* = 0.712, Fig. 2). Normal tissue from patients without cancer had similar 5-hmC compared to normal tissue from patients with synchronous CRC (*p* = 0.215).

### LINE-1 in polyps with synchronous CRC compared to those without synchronous CRC

**Fig. 3:**
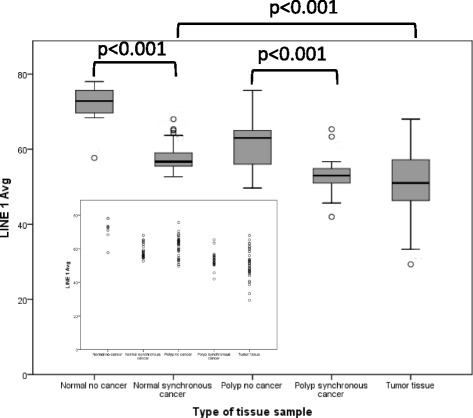
LINE-1 methylation levels in normal tissue from patients without CRC (*N* = 8), polyps from patients without and with CRC (*N* = 45, *N* = 32, respectively), normal tissue from patients with CRC (*N* = 32), and tumor tissue (*N* = 32). LINE-1 methylation in polyps from cancer patients was significantly lower than in polyps from non-cancer patients (*p* < 0.001). LINE-1 was also hypomethylated in tumor tissue compared to normal tissue (*p* < 0.001). Normal tissue from non-cancer patients showed the highest LINE-1 methylation levels (least hypomethylation) compared to polyps (*p* < 0.001), non-malignant tissue (*p* < 0.001), and tumor (*p* < 0.001). *Inset*: dot plot of LINE-1 methylation levels. All comparisons of means were performed by independent *t* test

The level of LINE-1 methylation in polyps from cancer patients (PC group) was decreased compared to polyps not associated with cancer (P group) (53.11 ± 4.48 vs 61.35 ± 6.02, *p* < 0.001, Fig. 3). LINE-1 levels did not differ between polyps with tubulovillous/villous features compared to patients with tubular adenomas (tubulovillous/villous 57.19 ± 7.6, tubular 56.32 ± 5.56, *p* = 0.78). LINE-1 levels also did not differ between polyps with and without CIMP positivity (57.65 ± 6.03 vs 58.57 ± 5.94, *p* = 0.539). The age of the PC group was somewhat older; therefore, we did a stratification subanalysis by age. Subanalysis of patients greater than age 60 found that LINE-1 remained significantly hypomethylated in the PC (*n* = 27; LINE-1 53.27 ± 3.99) versus the P group (*n* = 30; LINE-1 58.78 ± 5.46, *p* = 0.006).

### LINE-1 in normal tissue of patients with synchronous CRC compared to those without any associated neoplasia

We compared LINE-1 methylation in normal tissue of cancer and non-cancer patients to test whether LINE-1 methylation could predict a field defect in non-cancerous tissue. LINE-1 methylation was significantly lower in normal tissue from cancer patients compared to that from patients without any neoplasia who had a negative screening colonoscopy (58.07 ± 3.78 vs 71.50 ± 6.47, *p* < 0.001 by independent *t* test). As is depicted in Fig. 3, there was an overall field defect pattern in the LINE-1 methylation status with tumor tissue showing the lowest LINE-1 methylation (51.02 ± 8.48) and normal colonic mucosa of individuals without CRC showing the highest LINE-1 methylation levels (71.50 ± 6.47, *p* < 0.001 by independent *t* test).

### LINE-1 in polyps with synchronous CRC compared to tumor tissue

Significant differences were found in median hypomethylation of LINE-1 in patient-specific PC polyp and their respective tumor tissue by Wilcoxon signed rank test (*Z* = 2.05, *p* = 0.025). A modest trend of linear relation was found between hypomethylation of LINE-1 in a patient-specific PC polyp and hypomethylation of LINE-1 in their respective tumor tissue by Pearson’s correlation test (*r* = 0.387, *p* = 0.092).

## Discussion

Patients who undergo colonoscopy with negative findings of adenomatous polyps have very low rates of metachronous colorectal cancer [4]. Adenoma detection rate reports have shown that over time, most patients in screening programs will ultimately develop an adenomatous polyp [6]. Current approaches to defining patients in colonoscopy surveillance programs with advanced adenoma due for repeat colonoscopy are based on size, morphology (villous and high-grade dysplasia), and multiplicity. Under the current approach, the majority of patients identified above as high risk for metachronous neoplasia will not have recurrent advanced neoplasia when colonoscopy is repeated for surveillance [4–6]. Molecular approaches that might better characterize prognostic risk of synchronous and metachronous neoplasia could have implications to better target surveillance colonoscopy to those at highest risk for advanced neoplasia. We show in this study, for the first time, that LINE-1 methylation patterns in the polyps could be predictive of a synchronous CRC. We also found a trend toward LINE-1 hypomethylation in progression from normal tissue to adenoma to CRC, suggestive of a possible field defect. Our findings suggest that LINE-1 methylation status of adenomatous polyps or normal tissue obtained during surveillance colonoscopies could more effectively risk-stratify patients for development of synchronous CRC.

Epigenetic changes including hypomethylation have been implicated as early events in the neoplastic pathway to CRC development [10, 13, 16, 17, 21]. LINE-1 within CRC itself has been shown to be hypomethylated in tumors, more so than in normal surrounding mucosa [12]. In addition, reduced LINE-1 methylation has been shown to correlate with CRC stage [22]. Variation in hypomethylation has been described amongst adenomatous polyps [21]. Sunami et al. demonstrated early onset of LINE-1 demethylation early on in dysplasia development in colorectal epithelial cells [23]. Our findings show, similar to Sunami et al., that there was a trend toward hypomethylation in progression from normal tissue in the absence of neoplasia to adenoma to CRC which had the lowest levels of LINE-1 methylation.

Most importantly, in the current study, there appears to be significant differences in LINE-1 methylation patterns between polyps in those without a synchronous CRC (P group) versus those polyps with a synchronous CRC (PC group). The groups histologically were similar with a similar and low rate of villous features as would be expected. Subanalyses did not show that villous features impacted LINE-1 levels and that the difference between the PC and P groups in LINE-1 levels in adenoma was present in patients over 60 years of age. This study is novel in epigenetic assessment of similar histological precancerous adenoma as by LINE-1 informative of synchronous CRC. In addition, LINE-1 was increasingly hypomethylated in the normal tissue of those with CRC (PC group) versus those without associated neoplasia (P group).

Why LINE-1 hypomethylation appears to track better with synchronous CRC in comparison to 5-mC is not entirely clear. Although no differences in overall global methylation (5-mC) were seen in the synchronous versus non-synchronous cancer polyp groups, when all tissue types were combined, 5-methyl cytosine trended with LINE-1 promoter methylation levels. Using a cut point of <60% as hypomethylated for LINE-1 average promoter methylation, the relative global 5-mC levels were 0.297 with LINE-1 promoter methylation >60% and 0.097 with LINE-1 promoter methylation <60% (*p* = 0.055). LINE-1 hypomethylation is measured at specific gene promoters whereas global methylation includes gene bodies and intergenic areas, which may make LINE-1 more discriminatory of synchronous neoplasia.

There are several limitations to this study. LINE-1 may have different implications in the MSI pathway or in serrated lesions, and in this exploratory analysis, we did not investigate serrated lesions [24]. MSI testing was only done in those patients who met Bethesda clinical criteria at the time of specimen collection. Whether serrated pathway lesions show preferential hypomethylation based on synchronous neoplasia was not explored here. Inflammatory status of tumor, the specific molecular profile such as EGF-R status or MSI status, was not defined in this exploratory analysis. A strength of the study is that polyp groups based on morphology, occurrence in the right colon, gender, and CIMP status were all similar. We sought to address whether separate discrete lesions showed preferential hypomethylation, and we did not investigate if there was variation within the colon itself or whether there was an anatomical proximity effect of methylation in relation to location of the polyp to the synchronous CRC.

Our findings can be considered with others that have assessed LINE-1 in colorectal neoplasia as informative in CRC as a marker of field defect or cancerization [17, 22, 23, 25]. Similar to a previous finding by Shigaki et al. associating tobacco use with LINE-1 hypomethylation as a field defect for esophageal squamous cell carcinoma, environmental insults may contribute to LINE-1 hypomethylation as a field defect for CRC [26]. LINE-1 has also been shown to be hypomethylated in normal tissue in those with strong familial predisposition to CRC [27, 28]. Kamiyama et al. showed that LINE-1 hypomethylation in normal tissue predisposes to future increased risk for synchronous CRC [25]. Our key finding is preferential LINE-1 hypomethylation in adenomatous polyps with synchronous CRC in comparison to those adenoma in the absence of CRC. We believe this should lead to consideration of further work characterizing global methylation as a prognostic factor to portend future CRC risk within conventional adenoma cohorts.

## Conclusions

In summary, we show that low LINE-1 methylation level in conventional adenomas of the colorectum is associated with synchronous colorectal cancer. This work could lead to assessment of LINE-1 in premalignant adenoma to predict metachronous risk of advanced neoplasia. Global methylation markers such as LINE-1 could be informative in guiding surveillance by colonoscopy, which are currently not very accurate in defining patients at increased risk for advanced neoplasia at surveillance colonoscopy. A future extension of this data would be to investigate LINE-1 methylation status in patients with adenomas and determine if LINE-1 status could stratify metachronous risk upon surveillance colonoscopy for the development of neoplasia.
