# Erratum to: LINE-1 is preferentially hypomethylated within adenomatous polyps in the presence of synchronous colorectal cancer

**DOI:** 10.1186/s13148-017-0375-x

**Published:** 2017-07-27

**Authors:** Alice Chu Jiang, Lela Buckingham, William Barbanera, Amoah Yeboah Korang, Faraz Bishehsari, Joshua Melson

**Affiliations:** 1 Department of Internal Medicine, Rush University Medical Center, 1717 W Congress Parkway, 10 Kellogg, Chicago, IL 60612 USA; 2 Department of Pathology, Rush University Medical Center, 600 S. Paulina Street, 1014 AAC, Chicago, IL 60612 USA; 3 Division of Digestive Diseases, Department of Internal Medicine, Rush University Medical Center, 1725 West Harrison, Suite 206, Chicago, IL 60612 USA

## Erratum

After publication of the article [1] it was brought to our attention that one of the authors had their name misspelt. The fifth author should be “Faraz Bishehsari”.
